# Natural Killers Are Made Not Born: How to Exploit NK Cells in Lung Malignancies

**DOI:** 10.3389/fimmu.2017.00277

**Published:** 2017-03-13

**Authors:** Paolo Carrega, Guido Ferlazzo

**Affiliations:** ^1^Laboratory of Immunology and Biotherapy, Department of Human Pathology, University of Messina, Messina, Italy; ^2^Cell Factory Center, University of Messina, Messina, Italy; ^3^Cell Therapy Program, University Hospital Policlinico G.Martino, Messina, Italy; ^4^Division of Clinical Pathology, University Hospital Policlinico G.Martino, Messina, Italy

**Keywords:** natural killer cells, lung cancer, tumor microenvironment, cytotoxicity, cytokines

## Abstract

In recent years, progress has been made in the characterization of natural killer (NK) cells in lung malignancies, and we have now gained a better understanding of the frequency, localization, phenotype, and functional status of NK cells infiltrating these tumors. NK cell subset recruited in lung cancer is mainly capable of producing relevant cytokines rather than exerting direct cancer cell killing. Thus, the relevance of NK cells in tumor microenvironment might also go beyond the killing of tumor cells, being NK cells endowed with regulatory functions toward an ample array of immune effectors. Nevertheless, boosting their cytotoxic functions and redirecting the migration of cytotoxic NK cell subset to the tumor site might open new therapeutic avenues for lung cancer. Also, we believe that a deeper investigation into the impact of both conventional (e.g., chemotherapy) or new therapies (e.g., anti-immune checkpoints mAbs) on NK cell homeostasis in lung cancer patients is now required.

Several lines of evidence support the notion that natural killer (NK) cells play an important role in the control of tumor growth. Notably, along with the large array of results derived from experimental mouse models, a reduced NK cell lytic activity has been associated in human with an increased risk of cancer. Nevertheless, only a very limited number of NK cells can be detected within the majority of human solid tumors.

The discovery that NK cell can easily recognize and lyse tumor cells, immediately translated into hope that NK cells might find a place in the clinic as therapeutic tools. Extensive efforts have been made to exploit NK cells into the clinic and many of these attempts have mainly relayed on knowledge obtained by studying peripheral blood (PB) NK cells. However, the environmental context and cytokine milieu in which NK cells are present, and perceive potential activating signals, may strongly impact on their ultimate effector activity, thus offering one of the possible explanations of the inconsistent outcomes of the majority of NK cell-based therapies. As a matter of fact, several studies have aimed to investigate the biology of NK cells present within the neoplastic tissue and the clinical relevance of this infiltrate: in this context, lung cancer, the main cause of cancer-related mortality, represents a major topic for studying the anti-tumoral role of NK cells.

## NK Cells in Lung Cancer and Their Clinical Significance

The impact of NK cells as a potential prognostic marker in resected NSCLC still remains an open question. Although some early studies have demonstrated that the presence of NK cells in the immune infiltrate was associated with a lower risk of relapse and/or longer survival (1, 2), these analyses may now be considered incomplete or not exhaustive because of the use of markers not exclusively expressed by NK cells. Indeed, a more specific analysis of this aspect recently failed to find an association between the presence of NKp46^+^ NK cells at early stages of the disease with the clinical outcome (3). However, another recent report showed that the number of NK cell infiltrating lung cancer tissue (detected as CD56^+^CD16^+^ cells) positively correlated to the survival of lung cancer patients (4).

Although the clinical significance of NK cells in lung cancers seems to require more work, our knowledge about the features of NK cells infiltrating NSCLC has expanded considerably in recent years. A certain consensus has been reached regarding the evidence that NK cells associated to the immune infiltrate of NSCLC are quite different from those normally found in the non-tumor areas of the lungs (5–7), since normal lungs are mainly populated by CD56^dim^ NK cells, whereas tumors are mainly enriched in CD56^bright^ NK cell subset. Overall, the NK cell population found within NSCLC has been described as displaying profound alterations in the expression of relevant NK cell receptors, more specifically, downregulation of activating (NKp30, NKp80, CD16, DNAM-1) and inhibitory (ILT-2 and KIRs) receptors, as well as upregulation of NKG2A when compared to the normal counterpart (3). Functionally, intratumoral NK cells displayed impairments in the ability to both degranulate (8) and produce IFNγ (3) in response to classical NK cell targets, and they may rather acquire a proangiogenic phenotype due to the tumor microenvironment (TM) (9).

Nevertheless, these conclusions were mainly drawn by considering the NSCLC-infiltrating NK cells as a whole population. Recent reports suggested that expression of markers such as CD69, CD103, and CD49a can distinguish tissue resident from circulating NK cells (10). These advances indicate the necessity to revise our previous data and call for a thorough investigation of the various NK cell subsets infiltrating lung tumors. In this regard, high levels of CD69 have been observed on intratumoral NK cells (8) (interpreted as activation marker, at that time) when compared to matched non-tumoral lung tissues, thus suggesting that a substantial amount of NK cells may be actually retained within the TM upon migration (or alternatively upon *in situ* development). Along with CD69, CD103 has also been found to be expressed on a subset of NKp46^+^ cells within the TM (although potentially co-expressed by ieILC1) [(11) and personal observations by P. Carrega]. These observations propel new questions about the specific contribution of tissue-resident NK cells in lung tumor immunosurveillance as well as about their activity in already established tumors.

A relative abundance of CD56^bright^ NK cells was also observed in pleural effusions (PEs) from different type of primary and metastatic tumors. In contrast with NK cells isolated from solid lung cancer tissues, PE-NK cells express normal levels of both main activating receptors and MHC Class I-specific inhibitory receptors and they rapidly release cytokines upon exposure to neoplastic cells (12). These data further confirm how the microenvironment and cytokine milieu of lung cancer can exert a strong influence on NK cell effector activities (13, 14).

## Accumulation of CD56^bright^ NK Cells at the Tumor Site

**Figure 1:**
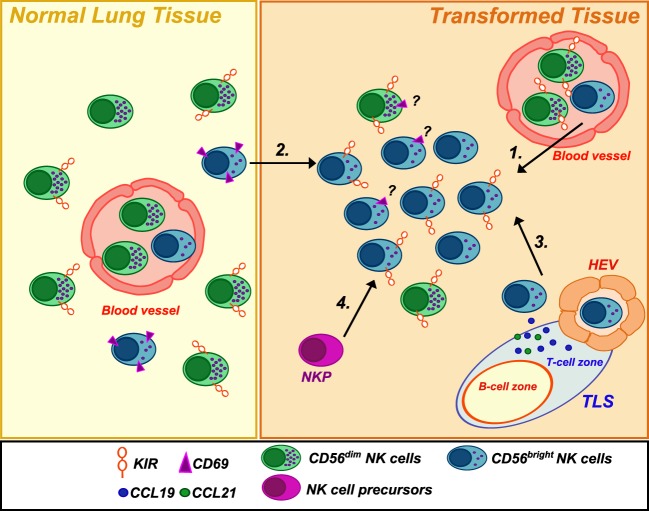
**Natural killer (NK) cell subsets in healthy and neoplastic lung tissues**. Human healthy lung tissues are mainly populated by CD56^dim^CD16^+^ NK cells but also present a small subset of CD56^bright^ NK cells expressing CD69, a marker of tissue-residency. Conversely, NSCLC tissues were found to be infiltrated by an NK cell population highly enriched in CD56^bright^CD16^neg^Perforin^low/neg^KIR^+^ cells. The reasons for such an accumulation are not clear. They may derive from (1) extravasation of PB NK cells from newly formed blood vessels and/or (2) migration of CD56^bright^ NK cells from the adjacent normal lung tissue in response to upregulated chemokines (CXCL9/CXCL10) in the neoplastic tissues. Moreover, (3) they may be recruited by chemokines (such as CCL19/CCL21) expressed in tertiary lymphoid structures or a lymphoid-like stroma *via* high endothelial venules (HEV), which represent a new gateway for lymphocyte entry into the tumor. Finally, (4) a local expansion from NK cell precursors (NKP) (21)/immature NK cells cannot be excluded. Noteworthy, the ratio of tissue-resident versus circulating/non-residing NK cells in neoplastic tissue has so far not sufficiently been deeply investigated.

Overall, current data show that NK cells are very rare within human NSCLC, and this evidence is in accordance with parallel observations in other solid tumors. Similarly to other tumors, NSCLC-infiltrating NK cells resemble PB-CD56^bright^ in their phenotype. These data raise various questions about the actual function of this “regulatory” NK cell subset at the tumor site. Primarily, whether the enrichment of CD56^bright^ NK cells in lung tumors represents a preferential recruitment of these cells from PB or adjacent tissues or rather a local expansion of immature NK cells within the tumor. Recent findings have revealed that tumor microenvironment may play a role in this specific accumulation. In particular, comparison of gene expression data between neoplastic and healthy lung tissues showed a chemokine switch (occurring upon neoplastic transformation) that is in agreement with the accumulation of non-cytotoxic CD56^bright^ NK cells recruited from PB (6). Specifically, variations in the tumor tissues involved a significant downregulation of CXCL2 that can selectively attract CD56^dim^ NK cells and, vice versa, an upregulation of chemokines specific for CCR7 and CXCR3 receptors (i.e., CCL19, CXCL9, and CXCL10), which are, on the contrary, preferentially expressed by CD56^bright^ NK cells. This might represent a further mechanism of cancer immunoediting with implications for both immunosurveillance and tumor escape from NK cell attack. Remarkably, also breast cancer, another tumor type characterized by enrichment in non-cytotoxic CD56^bright^ NK cells, displayed upregulation of genes coding for chemokines attracting this subset, when compared with gene expression profile of healthy breast tissues. Since NSCLC are often associated with the presence of intratumoral tertiary lymphoid structures (15), it is conceivable that these ectopic lymphoid tissues, as well as the establishment of a lymphoid-like stroma within the tumor, might drive the expression of chemokines normally secreted in secondary lymphoid organs (CCL19, CCL21, etc.) and, therefore, preferentially attract CD56^bright^ non-cytotoxic NK cells at the tumor site (Figure 1). An experimental approach employing the use of humanized mice xenograft models in which the xenograft develops in the context of a human immune system might potentially help in answering the question on which NK cell subset preferentially migrate to the neoplastic tissues and to shed further light on the mechanisms lying behind their migratory properties.

On the other hand, however, a local expansion of less mature NK cells in the tumor cannot be excluded. In agreement with this hypothesis, it has been reported that a significant amount of CD56^bright^perforin^neg^CD16^low^ NK cells infiltrating lung cancers (and breast cancer) showed upregulation of KIRs on their surface (8, 16). These observations support the hypothesis that TM might, at least in some cases, might favor the expansion of functional intermediate of the normal differentiation between the CD56^bright^ and CD56^dim^ human NK-cell subsets (17, 18). Thus, as previously shown in *in vitro* studies (17), CD56^bright^ NK cells might locally proliferate and eventually differentiate into the cytotoxic counterpart under adequate stimuli obtained within TM.

It is noteworthy that the analysis of perforin expression results critical in this context for identifying the two main distinct subsets of NK cells, since the intratumoral CD56^bright^CD16^neg^ NK cell phenotype could also represents the result of an activation-induced upregulation of CD56 and concomitant cleavage of CD16 by the ADAM17 protease (19, 20). Finally, we cannot exclude the possibility that tumor-infiltrating NK cells can originate from NK cell progenitors migrated to the tumor site (Figure 1). This hypothesis is supported by the recent discovery, in various human adult peripheral tissues, of an NK cell-restricted precursor (defined as Lin^neg^ CD34^+^ CD38^+^ CD123^neg^ CD45RA^+^ CD7^+^ CD10^+^ CD127^neg^) able to give rise to both CD56^dim^ and CD56^bright^ NK cells *in vitro* and *in vivo* and expressing high levels of CD62L on their surface (21).

## Functions of NK Cells in NSCLC Microenvironment

Collectively, we know relatively little about the factors that regulate NK cell response under different physio-pathological conditions; therefore, the potential activity of NK cells in lung cancer microenvironment can just be speculative. The notion that CD56^bright^ NK cells are a subset endowed with immunoregulatory properties is now in general accepted: this is related to their ability to secrete cytokines able to influence innate immunity as well as to their ability to assist in the polarization of primary adaptive immune responses in secondary lymphoid organs. However, in the context of human cancers, they may even exert an immune suppressive function. As a matter of fact, the CD56^bright^ CD16^low^KIR^+/−^ phenotype found in lung cancers is also reminiscent of NK cells present in decidua tissue and it might be induced upon NK cell entry into a tumor microenvironment expressing substantial TGFβ (22). Also, results from a recent investigation in melanoma are in line with a potential regulatory role by CD56^bright^ NK cells (23). According to this study, blood CD56^bright^CD16^neg^ NK cells express higher levels of ectoenzymes than CD56^dim^ NK cells; hence, they may act as “regulatory cells” by inhibiting autologous CD4^+^ T cell proliferation. This activity is mediated by production of adenosine, with a pivotal role played by CD38. Moreover, upon proper activation, CD56^bright^ NK cells are equally, if not more, cytotoxic than CD56^dim^ NK cells against autologous activated CD4^+^ T cells (24). This observation may be of interest in NSCLC, where NK cells were mainly found in the tumor stroma, in close contact with other leukocytes (8). It should be noted that also NK cells from PE, upon IL-2 activation, gain a strong cytolitic activity against tumor cells (12), even stronger than PB-NK cells undergoing the same activation. These findings raise new questions about a suitable therapeutic exploitation of NK cells: more specifically, about the possibility to boost cytotoxic function of CD56^bright^ NK cells against cancer cells while avoiding elimination of activated autologous CD4^+^ T cells.

As a whole, however, few *in vivo* data have so far been collected in support of an immunosuppressive role of NK cells within tumor tissues and in lung cancer in particular. Nevertheless, this remains an interesting and poorly explored field of investigation.

On the other hand, NK cells have been implicated in the response to tumors also because of their abundant release of IFNγ. Experimental animal studies highlighted the importance of NK cell-mediated secretion of IFNγ in tumor rejection. In particular, the initial expression of IFNγ by NK cells may induce expression of CXCR3 ligands and, in turn, contribute to further accumulation of NK cells and tumor rejection (25). Tu et al. showed a pivotal role for CD160 and IFNγ secretion in controlling tumor growth by NK cells (26). An early release of IFNγ may be of relevance also for upregulating co-stimulatory molecules on dendritic cell (DC) surface, endowing DCs with a more potent triggering potential for tumor-specific CD8^+^ T cells (27), but also, as highlighted below, for the expression of ligands of immune inhibitory receptors.

## Role of NK Cells in Conventional and Unconventional Anticancer Therapy

The role of NK cells in the conventional therapy of lung cancer is only partially known; in addition, the possible modifications of NK cell activities and homeostasis upon administration of therapies currently used for lung cancers, ranging from conventional chemotherapy to anti-PD1 blocking mAbs, have been thus far scarcely investigated. Nevertheless, various evidences support the notion that beneficial effects of conventional therapies may also be mediated as “off-target” mechanisms by this innate immune cell population. For example, both cisplatin (28) and gemcitabine treatments (29) induce upregulation of NKG2D ligands on cancer cells. Similarly, also inhibitors of the MEK/ERK signaling pathway can upregulate the expression of ULBP2, one of the ligand of NKG2D, by inducing demethylation of the ULBP2 promoter (30). Moreover, NK cells may contribute to the efficacy of targeted therapies, such as cancer-associated fibroblast-targeted therapy used in combination with DC-based vaccines (31), or agents targeting EGFR, such as erlotinib (32) and necitumumab. The latter has been approved as a first-line treatment for advanced squamous NSCLC, the less immunogenic type of NSCLC tumor. Interestingly, the necitumumab–EGFR complex can induce ADCC by various types of immune system cells, such as NK lymphocytes (33).

In light of the therapeutic benefit obtained with immune checkpoint inhibitors, such as mAbs blocking programed cell death protein 1 (PD1) pathway, it would be interesting to further investigate the effects of these classes of drugs on NK cell compartment. Anti-PD1 (nivolumab and pembrolizumab), anti-KIR (lirilumab), and anti-NKG2A (monalizumab) may act by removing a brake on the effector functions of NK cells. Anti-PD1 agents have been already approved for the treatment of advanced NSCLC and, very recently, have shown promising results when used in the neoadjuvant setting (34). Anti-PD1 therapy might unleash the cytotoxic potential of activated NK cells expressing PD1 (35), thus contributing to the anticancer activities of this drug. On the other hand, NK cells might also be detrimental for avoiding PD1 blocking since IFNγ derived from tumor-infiltrating NK cells may further increase the expression of the ligand PD-L1 in the tumor microenvironment.

While we are now accomplishing groundbreaking results by anti-PD1 agents, anti-KIR agents are currently in phase II; nonetheless, they may be important in various settings, in neoadjuvant and advanced settings but also as adjuvant therapy in order to reduce risks of recurrence. Along with KIRs, targeting of the inhibitory receptor NKG2A (monalizumab) is currently under investigation as single agent or in combination with other agents in different types of solid tumors (36). The rationale for the use of this agent in lung cancer is also supported by the higher expression levels of NKG2A in intratumoral NK cells if compared to NK cells in normal lung tissues (6, 8). Another therapy targeting the inhibitory receptor LAG3 (BMS-986016), expressed on lymphocytes and NK cells, is also currently investigated (NCT01968109) alone and in combination with nivolumab in subjects with select advanced (metastatic and/or unresectable) solid tumors. Further increasing the number of targetable checkpoints on NK cells, recent preclinical data demonstrated the utility of blocking CD96 for enhancing NK cell activity against experimental and spontaneous metastases (37). Finally, targeting costimulatory receptors such as CD137 (4-1BB), expressed on activated NK cells and cytotoxic T cells, may represent another option, especially in combination with mAbs targeting TAA, as shown for head and neck cancer patients (38).

## Conclusion and Perspective

**Figure 2:**
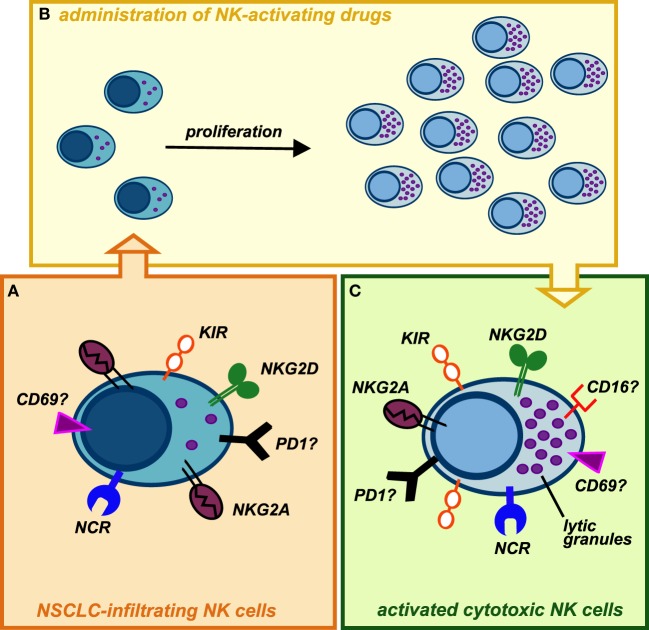
**Possible conversion of lung cancer-infiltrating natural killer (NK) cells into highly cytotoxic killer cells upon administration of NK-activating drugs**. **(A)** NK cells isolated from NSCLC tissues display phenotypic features similar to those of peripheral blood (PB) CD56^bright^ NK cells, with the exception of KIRs, that can be detectable on these cells. Similarly to PB CD56^bright^ NK cells, they are endowed with high proliferative potential but impaired in their cytotoxic capability. In addition, it is not clear whether NK cells infiltrating human lung tumors express other immune checkpoints (such as PD-1), which might further contribute to their poor cytolytic activity. **(B)** Upon activation with stimulating cytokines (recombinant IL-15, IL-2) or mAbs-blocking inhibitory receptors (e.g., anti-NKG2A, which is highly expressed on these cells, or anti-PD1), intratumoral NK cells may proliferate *in situ* and modify their phenotype acquiring strong cytolytic capabilities against cancer cells. **(C)** Administration of cytokines or other NK-activating drugs, currently available, can upregulate the expression of cytolytic granules on expanded tumor-infiltrating NK cells but also induce higher levels of KIR inhibitory receptors: this might be circumvented by administration of anti-KIRs mAbs. It remains to be investigated whether *in vivo* stimulation of lung cancer-infiltrating NK cells can upregulate on these cells the expression of activating receptors (e.g., CD16), immune checkpoints, or markers of tissue residency.

Despite knowledge on NK cell biology significantly increased in these last years, we surprisingly still know relatively little about their functions in TM. This is particularly astonishing considering that NK cell discovery was related to their strong ability to recognize and kill cancer cells without prior sensitization. Clearly, further knowledge, particularly in human setting, is required to better exploit this abundant innate lymphocyte population in lung cancer treatment. However, recent evidences indicate that CD56^bright^ NK cell population infiltrating lung cancers, endowed with low ability to kill cancer cells, can spontaneously acquire KIR receptors and thus further differentiate, suggesting that they might convert in cytotoxic killer cells. Remarkably, CD56^bright^ NK cells, the main NK cell subset infiltrating lung cancer, can undergo robust proliferation upon appropriate cytokine stimulation. At the same time, factors able to efficiently activate NK cells, induce their proliferation and to terminally differentiate them into highly cytotoxic NK cells are currently available as clinical-grade agents (e.g., IL-15 and/or mAbs blocking NK inhibitory receptors) (Figure 2). Finally, although few data are currently available, we recently started to shed some light on the mechanisms ruling he recruitment of NK cells within lung cancer tissues. The use of factors able to modify lung cancer expression of chemokines involved in NK cell recruitment could represent an interesting therapeutic avenue.

While further basic investigation on the biology of lung cancer NK cells is clearly required, we recently gained a better insight in this field. Novel immunotherapeutic strategies for lung cancers, conceivably in combination with established anticancer therapies, should now aim at increasing the amount of cytotoxic NK cells in lung cancers: that could be achieved not only recruiting them from PB but also driving their proliferation and differentiation within TM.

## Author Contributions

PC and GF equally contributed in writing of this manuscript.

## Conflict of Interest Statement

The authors declare that the research was conducted in the absence of any commercial or financial relationships that could be construed as a potential conflict of interest.
